# Iron Removal from Metallurgical Grade Silicon Melts Using Synthetic Slags and Oxygen Injection

**DOI:** 10.3390/ma15176042

**Published:** 2022-09-01

**Authors:** Xiao Long, Wenbo Luo, Guohong Lu, Falou Chen, Xiaoning Zheng, Xingfan Zhao, Shaolei Long

**Affiliations:** 1 School of Materials and Energy Engineering, Guizhou Institute of Technology, Guiyang 550003, China; 2 Yunnan Yongchang Silicon Industry Co., Ltd., Baoshan 678300, China; 3 Yunnan Longling County Longshan Silicon Co., Ltd., Baoshan 678300, China; 4 Zhejiang Xin’an Chemical Group Co., Ltd., Jiande 311600, China; 5 Longling Pengyue Science and Technology Consulting Service Co., Ltd., Baoshan 678300, China

**Keywords:** synthetic slag, metallurgical-grade silicon, refining, Fe removal, oxygen injection

## Abstract

Novel SiO_2_-CaO-CaF_2_-R_2_O-MgO based synthetic slags (R_2_O represents alkali metal oxides) with varied binary basicity values were used with oxygen injection to refine silicon melts and remove Fe from metallurgical-grade silicon. Silicon samples and slags at the silicon-slag interfaces were obtained during refinement. The compositions of the silicon samples were analyzed, and the quenched slag samples and mild cooling slags from the final crucible were inspected using scanning electron microscopy and energy dispersive X-ray spectroscopy. After 15 min of refinement, the Fe removal rate ranged from 52.3 to 60.1 wt%. During the refining process, the Fe-concentrated phase formed within the silicon droplets and was then transferred to the silicon-slag interfaces and wetted with slags. The Fe-concentrated phase at the silicon-slag interface can dissolve directly in the slags. It can also be transferred into the slag phase in the form of droplets, which can be affected by the binary basicity of the slags. Ti removal demonstrated a similar mechanism. Fe-bearing crystals were not detected in the quenched slag samples obtained during refinement, while complex Fe-bearing phases were detected in the final slag. This study demonstrates Fe removal from metallurgical-grade Si using slag refining methods and reveals the removal mechanism during the refinement.

## 1. Introduction

Metallurgical-grade silicon (MGS) is an essential raw material for the electronic and organic chemical industries. It is typically produced by reducing silica ore (using carbonaceous materials) at high temperatures in electric arc furnaces. During the manufacture of MGS, gangue minerals, such as iron oxides are also reduced, increasing the concentration of impurity elements in silicon melts [1,2,3,4].

Fe is an essential impurity in metallurgical-grade Si. As iron oxides can be easily reduced by carbon at high temperatures, most of the Fe content in raw materials can be transferred into silicon melts. Currently, ladle refinement is typically required to remove parts of the impurity elements in Si melts after tapping [5,6]. Owing to its simplicity, oxygen-bottom blowing is the most frequently adopted method for refining raw MGS melts. Commercial practices indicate that impurity elements, such as Al and Ca can be oxidized and removed by forming top slags during oxygen injection refinement processes. However, elements with a much weaker affinity for oxygen, such as Fe, Mn, Cr, and V (as indicated by Ellingham diagrams), cannot be effectively oxidized [7]. Numerous approaches have been reported on using slags to refine silicon melts, such as the removal of Al, Ca, and B by slag refining. Related slag systems, such as CaO-SiO_2_, CaO-SiO_2_-CaF_2_, and CaO-SiO_2_-MgO, were investigated [8,9,10,11]. However, hardly any references have been reported on removing Fe from silicon melts using slag refinements, because the impurity removal mechanism has been considered to be the oxidation of those elements by SiO_2_ in slags or oxygen injected in silicon (then absorbed by slags). However, Fe cannot be oxidized by SiO_2_ to form steady FeO based on thermodynamics analysis [7]. Therefore, Fe has not been considered removable using ordinary slag oxidizing refinement methods.

To purify MGS for use in applications including monocrystalline and polycrystalline silicon manufacturing, Fe in raw MGS must be effectively removed. Numerous studies have discussed the removal of Fe from raw MGS, most of which focus on methods including acid leaching after grinding MGS into powders, directional solidification, external fields, or liquation refining (using alloys to absorb impurities). A few of these methods have proven to be effective for impurity removal [12,13,14,15,16,17,18,19,20,21,22,23]. However, simple grade control of raw materials, resulting in lower impurity inputs, is still the most common method utilized for refinement in the MGS manufacturing industry.

As supplies of high-quality silica ore are low in China, the efficient utilization of high Fe content silica ore is of critical importance. In our previous study on Ti removal from MGS, high basicity index slags and carbon dioxide injection were used, and the Fe concentration within silicon droplets was observed during refinement. The concentrated impurity phase wetted with slag at the silicon-slag interface demonstrated a potential method for Fe removal [24]. Based on previous results, novel SiO_2_-CaO-CaF_2_-R_2_O-MgO based synthetic slags (R_2_O represents alkali metal oxides) with varied binary basicity values were utilized in this study to refine high Fe content MGS. Related refining experiments were conducted to prove the feasibility of Fe removal from metallurgical-grade Si using slag refining methods and to discuss the Fe removal process.

## 2. Materials and Methods

### 2.1. Materials and Experimental Process

A high-Fe MGS sample was used as the basis of this study. The contents of typical impurity elements in this silicon sample are listed in Table 1 (measured using an ICP-MS, PerkinElmer NexION 2000, Waltham, MA, USA). The compositions and basic physical properties (viscosity and melting temperature) of the three synthetic slags with different binary basicity values are listed in Table 2. The viscosity of the slags was measured at 1673 K using a rotational viscometer with a graphite bob (15 mm diameter) and a graphite crucible (60 mm inner diameter) to hold the liquid slags. The melting temperatures of the initial slags were measured using the hemispherical point method, which has been described in previous studies [25,26].

Slags for refinement were prepared using analytical reagents (CaCO_3_, SiO_2_, Na_2_CO_3_, Li_2_CO_3_, MgO, Al_2_O_3_, and CaF_2_), high-purity graphite powder, and carbon black (to provide C_free_). The slag samples were first pre-melted in high-purity graphite crucibles in resistance furnaces (except for graphite powder and carbon black). They were then cooled in air (a glassy state formed and no crystals were detected using XRD and optical microscopy) and ground with graphite powder and carbon black. The particle sizes of the prepared synthetic slags for experiments were below 74 µm. For each experiment, a silicon lump weighing 250 g was placed with 30 g of ground slag powder in a high-purity magnesia crucible (inner diameter 40 mm). These were melted in a resistance furnace using MoSi_2_ heating elements at a holding temperature of 1773 K. Oxygen was injected into the silicon melts at a rate of 50 mL/min through a high-purity quartz tube with an inner diameter of 3 mm (immersed into silicon melts from the top of the crucible). In order to understand the iron removal mechanism, silicon and slag samples were acquired using another quartz tube after 5, 10, and 15 min of oxygen blowing (oxygen blowing was suspended before sample acquisitions). Samples from the silicon-slag interface were rapidly quenched to preserve the emulsification detail, which had been detected in our previous work [24]. After 15 min of refinement, the crucible temperature was maintained at 1773 K for 5 min and then cooled in the furnace. Melting of the quartz tube for oxygen injection was not observed after refining. The refining experiments were repeated three times to verify the authenticity of the results. The refining reactions in this study are exothermic, and they could cause a temperature fluctuation in silicon melts during the refinement process. Thus, a B-type thermocouple placed at the bottom of the crucible was used to take temperature measurements. During sample acquisition, the temperature of the silicon melts was directly measured using a B-type thermocouple immersed in the silicon melts (the thermocouple was protected using a magnesia tube and preheated in the furnace). The results indicated that the temperature differences during the refinements in this study were less than 20 K.

### 2.2. Measurements

An ICP-MS (PerkinElmer, NexION 2000, Waltham, MA, USA) was used to measure the impurity elements of the acquired silicon samples (0.25 g of each silicon sample, progressively digested using hydrofluoric acid, nitric acid, perchloric acid, and hydrochloric acid under atmospheric pressure). Furthermore, scanning electron microscopy (SEM, FEI Company, Nova Nanosem 450, Hillsboro, OR, USA) and energy dispersive X-ray spectroscopy (EDS) were used to reveal the micro-morphology features and the corresponding compositional information of the obtained samples. The samples for SEM-EDS inspection were prepared by mounting slag and silicon lumps in resin, polishing with an alumina suspension, and splutter-coating them with gold. The yield of silicon after refining was calculated using its weight loss (silicon lump obtained in the crucible cooling in furnace; the weight of silicon samples obtained during refining was also counted).

## 3. Results and Discussion

### 3.1. Iron Removal Efficiency

**Figure 1:**
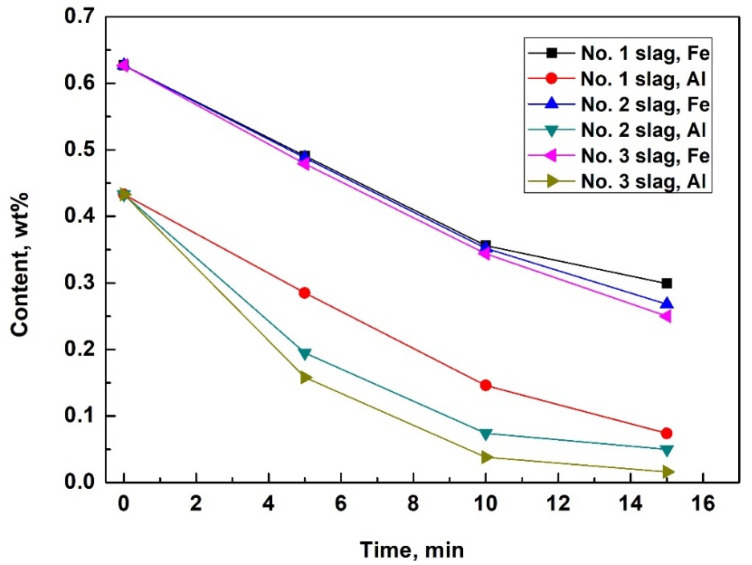
Fe and Al content of MGS during refinement using slags with three different binary basicities (0.4 for No. 1 slag, 0.5 for No. 2 slag, and 0.6 for No. 3 slag).

Some of the measured impurity elements of the silicon samples obtained during refinement are outlined in Table 3 and Figure 1. Slags No. 1, 2, and 3 have basicities of 0.4, 0.5, and 0.6, respectively. The results indicate that the Fe content of the silicon melts decreased significantly. After 15 min of refining, the Fe removal rates were 52.3% for slag No. 1, 58.1% for slag No. 2, and 60.1% for slag No. 3. Slags with a higher basicity value and lower viscosity exhibited slightly higher Fe and Ti removal rates after 15 min of refinement. In addition to Fe, other impurity elements such as Al and Ca also decreased to some degree. However, compared with the removal rates for Fe and Ti, slag compositions were more likely to affect the Al and Ca removal rates. Slags with higher basicity values and lower viscosities resulted in higher Al removal rates and lower Ca removal rates. CaO is a basic oxide, and increased basicity can restrict the dissolution of CaO into slags after the oxidation of Ca. This could explain why a higher basicity decreased the Ca removal rate. Al_2_O_3_ is an amphoteric oxide, and higher basicity values provide lower slag viscosities and better dynamic conditions for Al_2_O_3_ dissolution. This could result in a higher Al removal rate when slags with higher basicity values are used. The different effects of the slag composition on the removal rates of Fe, Ti, Al, and Ca were caused by their different removal mechanisms, which will be discussed later in this report.

The calculated yields of silicon after refining are 98.6%, 98.1%, and 98.0% for No. 1, No. 2, and No. 3 slag, respectively. Repetition of the experiments exhibited no obvious statistical differences on the impurity removal efficiencies and silicon yields.

### 3.2. Transferring of Iron from Silicon to Slag

**Figure 2:**
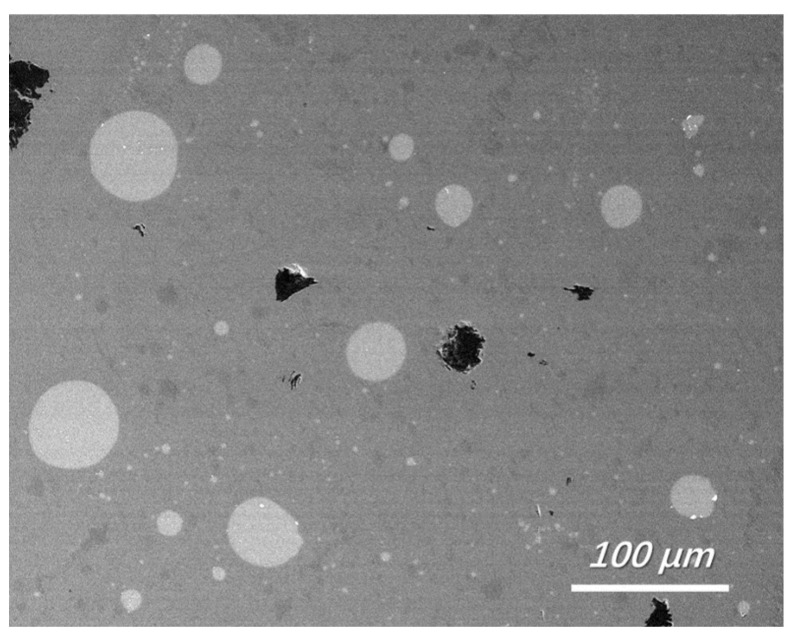
Feature of quenched slag near the silicon-slag interface (backscattered electron image, No. 1 slag with a refining time of 5 min).

Slag at the silicon-slag interface was obtained and rapidly quenched. Typical features of the quenched slags are shown in Figure 2. Similar to results demonstrated in other research on Ti removal [24], numerous silicon droplets were observed in the quenched slag in this study. The emulsification of the silicon-slag system can improve the dynamic conditions of the Fe removal process. Table 4 shows the typical size distribution of silicon droplets in the slag samples obtained at the silicon-slag interface (samples were obtained and quenched after 5 min of refinement). The results indicate that a higher basicity of the initial slag tends to limit the emulsification of the silicon-slag system during refining, decreasing the interfacial area of the silicon-slag system and deteriorating the dynamic conditions for Fe removal.

However, the increased basicity of slags resulted in a slightly higher Fe removal rate (as shown in Figure 1), because the Fe in the silicon melts dissolved into slags at silicon-slag interfaces and directly transferred into the slag as droplets of Fe-concentrated phases. The latter procedure can be intensified in slags with higher basicities and lower viscosities, which will be discussed later.

**Figure 3:**
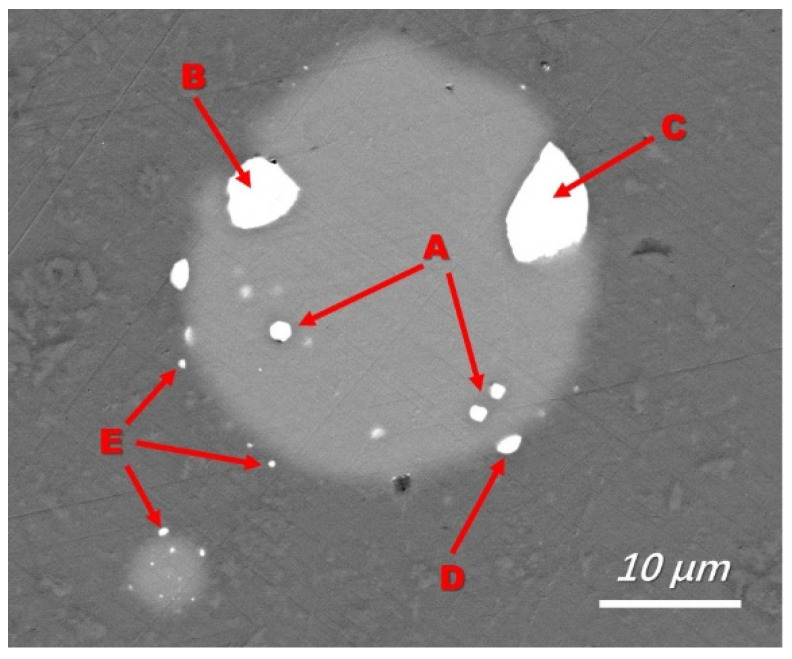
A typical Fe-concentrated phase in silicon droplets (backscattered electron image, No 1 slag with a refining time of 5 min). A: Fe-concentrated phase formed within the silicon drop; B: Fe-concentrated phase transferred to silicon-slag interface; C and D: Fe-concentrated phase wetted with slag phase and dissolved in slag; E: undissolved Fe-concentrated phase transferred into the slag.

**Figure 4:**
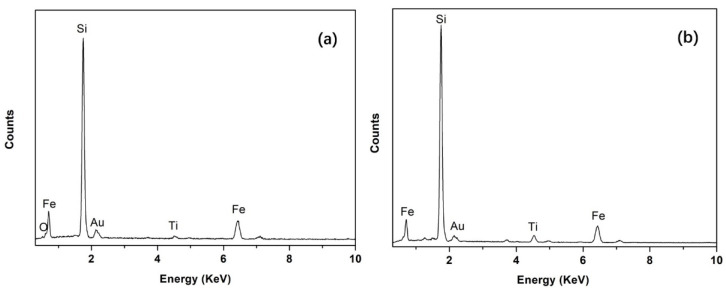
Typical EDS analysis of the bright Fe-concentrated phase at the silicon-slag interface (**a**) and within the silicon droplet (**b**).

To reveal the Fe removal mechanism, a typical backscattered electron image of silicon droplets with a detailed morphology is shown in Figure 3 (sample obtained and quenched after 5 min of refining), where a silicon droplet of approximately 15 µm in diameter is displayed. Typical EDS analyses of the bright phase at the silicon-slag interface and within the droplet (Figure 4) demonstrated that Fe and Ti were concentrated. The Fe-concentrated phase was wetted with slags at the silicon-slag interface, which indicated that in this case, instead of solidification segregation, the Fe-concentrated phase formed during refinement. Table 5 shows that compared with impurity phases at the silicon-slag interface, the ones within the silicon droplet contain slightly higher content of Ti because parts of concentrated Ti had already been dissolved into slag phases.

**Figure 5:**
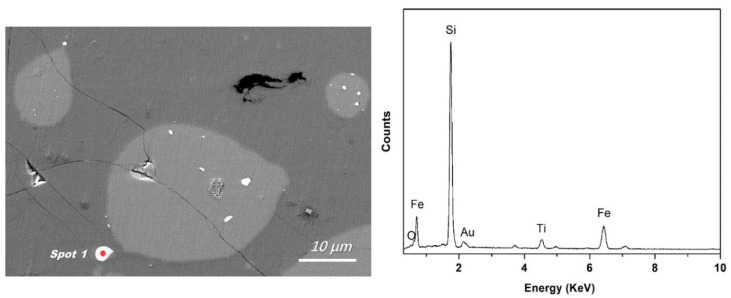
Typical morphology of an independent Fe-concentrated droplet in slag (at spot 1, backscattered electron image) and the corresponding EDS analysis at spot 1.

Area E in Figure 3 suggests that the Fe-concentrated phase could leave the silicon-slag interface and transfer directly into the slag. In the slag with a lower initial viscosity (slag No. 3), more apparent Fe-concentrated droplets were observed, as shown in Figure 5. Figure 5 also shows typical EDS analysis of this droplet. The EDS results in Figure 4 and Figure 5 are similar, indicating that the independent Fe-concentrated droplets in the slag originated from those at the silicon-slag interface. The independent Fe-concentrated droplets in different slags were investigated, and it was determined that a higher slag basicity tended to result in larger independent Fe-concentrated droplets in the slag.

**Figure 6:**
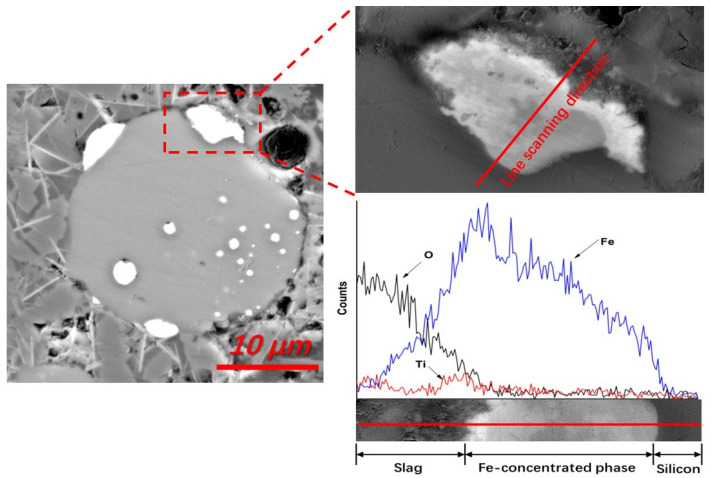
EDS line scanning of a typical Fe-concentrated phase at the silicon-slag interface, demonstrated by a Fe-rich boundary layer (backscattered electron image, No. 1 slag, sample cooling in furnace).

In addition to the Fe-concentrated droplets that were transferred directly from the silicon-slag interfaces to the slag, the Fe-concentrated phase also dissolved at the silicon-slag interface. The diffusion dynamic conditions of slags during refining were optimal, so no apparent Fe-concentrated boundary layer in the slags was detected in the quenched samples. However, as shown in Figure 6, this boundary layer can be detected in the slag samples from crucibles cooled in the furnace. As the viscosity of the slag could be increased and the Fe-diffusion capacity in the slag could be decreased with a slow decrease in temperature, a detectable Fe concentration boundary layer was observed using EDS line scanning. In addition to Fe, Ti was also concentrated in this boundary layer and in all Fe-concentrated phases to some degree, indicating the presence of a similar removal mechanism. From the statistical analysis of EDS, it was determined that the Fe and Ti contents of the Fe-concentrated phase that formed during refining ranged from 20.32 wt% to 34.56 wt%, and 0.55 wt% to 5.80 wt%, respectively. No apparent concentrations of Al and Ca were detected in the Fe-concentrated phase or this boundary layer, indicating that a different removal process occurs for these impurities.

**Figure 7:**
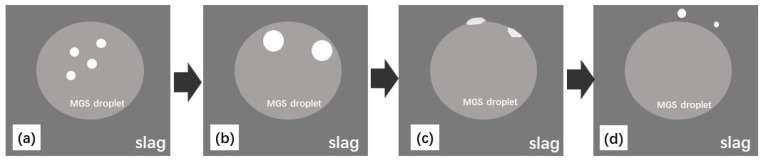
Schematic diagram of the Fe-concentrated phase of MGS transferred to slag; (**a**) Fe-concentrated phase formed within silicon droplets; (**b**) Fe-concentrated phase transferred to silicon-slag interface; (**c**) Fe-concentrated phase wetted with slag and dissolved; (**d**) undissolved Fe-concentrated phase transferred into slag.

Based on the previous discussion, the Fe removal procedure should follow four stages: (1) the Fe-concentrated phase is formed within the silicon droplets during refinement; (2) the Fe-concentrated phase is transferred to silicon-slag interfaces; (3) the Fe-concentrated phase is wetted with slag and dissolved; and (4) any undissolved Fe-concentrated phase is transferred directly into the slag. These steps were observed, as marked in Figure 3 and illustrated in Figure 7.

### 3.3. Existence of Fe in Refining Slag

**Figure 8:**
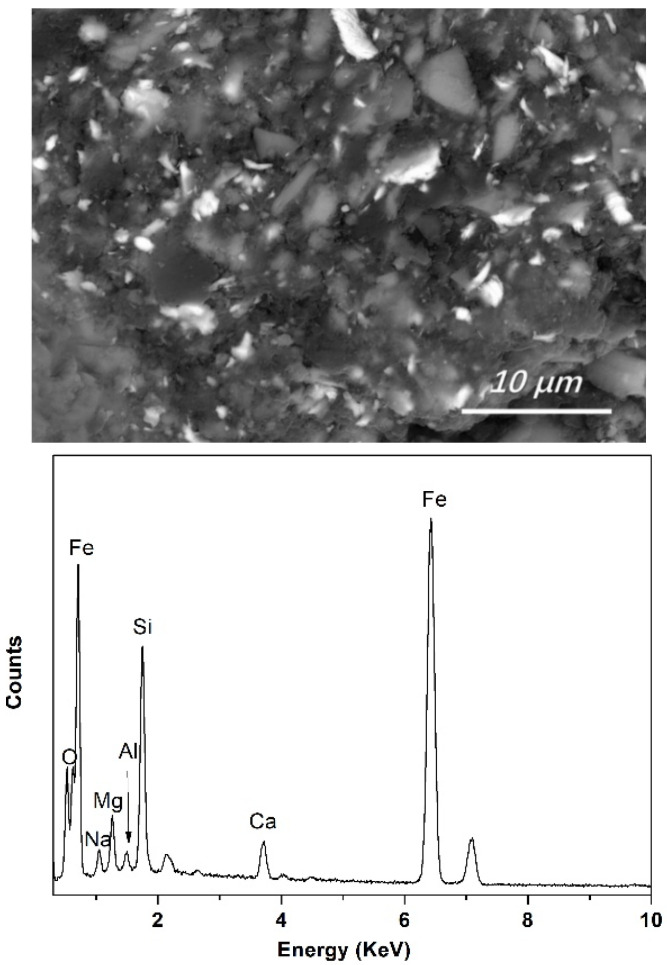
Typical morphology of Fe-concentrated phase in final slag and EDS analysis of the bright Fe-concentrated phase (backscattered electron image, No. 1 slag, sample cooling in furnace).

The Fe content in the final slags was very low and might not precipitate as crystals, so no Fe-containing crystals were detected using X-ray diffraction. However, clear Fe-concentrated phases were detected in the mild cooling slags using EDS. The typical morphology of the Fe-bearing phase in the slag (bright area in Figure 8) and the corresponding EDS results are shown in Figure 8. To avoid interference from the slag matrix, a low accelerating voltage of 15 kV was used during the EDS analysis. Instead of simple oxidates, the Fe-concentrated phase in the final slag (from crucible cooling in the furnace) was presented as a finely mixed compound with a complex composition of Fe, Si, Al, Mg, Na, Ca, and O. From statistical analysis, it was determined that the Fe content of the bright phase in the final slags ranged from 30 wt% to 55 wt%.

However, no apparent Fe-bearing crystals were detected (using SEM-EDS) in slags obtained near silicon-slag interfaces during refinement (quenched to preserve the slag structure at high temperatures). The results indicate that during refinement, instead of precipitating as Fe-bearing crystals, Fe steadily dissolved into liquid slags. This could precipitate as crystals after mild cooling. To avoid the transfer of dissolved Fe in slags to the silicon melts during the refinement process, further research is required to measure the Fe capacity of the slag and its influencing mechanisms during refinement.

During silicon refinements, Fe cannot be oxidized to form steady FeO and be transferred into the slag using slag refinements. However, our experiments revealed a novel Fe-removal mechanism. Instead of the direct formation of FeO by oxidation, Fe of raw MGS formed liquid impurity phases at the silicon-slag interface with complex compositions (mainly as Fe, Ti, Si, and O), and we detected the transfer of this phase from silicon to slag. More studies on the chemical potential and activity of Fe in this slag system are required.

Compared with approaches presented in other papers [13,14,15,16,17], such as acid leaching and directional solidification, the novel Fe-removal method in this study requires simpler facilities and can process large batches of Si melts directly after tapping with high efficiency, and no pretreatment of Si melts is required. Thus, the method presented in this work can be potentially applied in ladle refining to remove Fe from MGS effectively, and for the use with high-Fe silica ore.

## 4. Conclusions

In this study, novel SiO_2_-CaO-CaF_2_-R_2_O-MgO based synthetic slags with different binary basicity values and oxygen injections were used to refine high-Fe MGS melts. The results of this study prove Fe removal from metallurgical-grade Si is possible using slag refining methods. A new Fe-removal mechanism was also presented. The following conclusions were drawn:

1. Fe in the MGS can be removed using SiO_2_-CaO-CaF_2_-R_2_O-MgO based synthetic slags and oxygen injection. 52.3 wt% to 60.1 wt% of the Fe in the raw MGS can be removed after 15 min of refining. The Fe removal rate was slightly enhanced by increasing the binary basicity of the slag.
2. Under the conditions of this study, emulsification of the silicon-slag system was observed, which could be restricted by increased binary basicity value.
3. During refinement, Fe-concentrated phases formed within the silicon droplets were transferred to silicon-slag interfaces and then wetted with slags. The Fe-concentrated phases at the silicon-slag interfaces can dissolve into slags directly or transfer into the slags as Fe-concentrated droplets. All the Fe-concentrated phases contained different amounts of Ti. The Fe and Ti contents of the Fe-concentrated phase formed during refining ranged from 20.32 wt% to 34.56 wt%, and 0.55 wt% to 5.80 wt%, respectively.
4. Complex Fe-bearing phases (containing 30 wt% to 55 wt% of Fe) were detected in the final slag cooled in the furnace, but no Fe-bearing crystals were detected in the quenched slags obtained during refining.

The method presented in this study provided a new option to remove Fe from Si melts using slag refining, which is feasible in ladle refining for Fe-removal and for use with high-Fe silica ore. However, more researches on characteristics of the new slag system are required.

## Figures and Tables

**Table 1:** Content of typical impurity elements in silicon samples, wt%.

Fe	Al	Ca	Ti	P
0.627	0.433	0.079	0.062	0.007

**Table 2:** Composition (wt%) and physical properties of initial refining slags.

Slag No.	CaO	SiO_2_	CaF_2_	Al_2_O_3_	MgO	Na_2_O	Li_2_O	C_free_	T_m_, K	η_1673K_, Pa.s	CaO/SiO_2_
1	18	45	10	3	6	12	2	4	1453	0.287	0.4
2	21	42	10	3	6	12	2	4	1477	0.245	0.5
3	23.6	39.4	10	3	6	12	2	4	1498	0.188	0.6

**Table 3:** Content of typical impurity elements of silicon samples obtained during refinement, wt%.

Time, Min	No. 1 Slag				No. 2 Slag				No. 3 Slag			
	Fe	Al	Ca	Ti	Fe	Al	Ca	Ti	Fe	Al	Ca	Ti
Initial MGS	0.627	0.433	0.079	0.062	0.627	0.433	0.079	0.062	0.627	0.433	0.079	0.062
5	0.491	0.285	0.046	0.060	0.488	0.195	0.059	0.054	0.479	0.158	0.061	0.053
10	0.356	0.146	0.032	0.051	0.352	0.074	0.042	0.048	0.344	0.038	0.047	0.041
15	0.299	0.074	0.023	0.043	0.268	0.050	0.031	0.040	0.250	0.016	0.035	0.036

**Table 4:** Size distribution of silicon droplets/mm^2^.

Slag No. (Basicity)	1 (0.4)	2 (0.5)	3 (0.6)
Overall quantity	79	56	35
1 µm < diameter < 10 µm	52	25	17
10 µm < diameter < 100 µm	27	30	15
Diameter > 100 µm	0	1	3

**Table 5:** Composition of impurity phases in silicon droplet in Figure 3, wt%.

Location	Si	Ti	Fe	O
A	64.77	2.48	32.75	-
B	63.70	2.53	33.77	-
C	65.52	1.25	31.12	2.11

## Data Availability

Not applicable.
